# Inhibition of Hippocampal Synaptic Activity by ATP, Hypoxia or Oxygen-Glucose Deprivation Does Not Require CD73

**DOI:** 10.1371/journal.pone.0039772

**Published:** 2012-06-25

**Authors:** Dali Zhang, Wei Xiong, Stephanie Chu, Chao Sun, Benedict C. Albensi, Fiona E. Parkinson

**Affiliations:** 1 Department of Pharmacology and Therapeutics, University of Manitoba, Winnipeg, Manitoba, Canada; 2 Division of Neurodegenerative Disorders, St Boniface Hospital Research Centre, Winnipeg, Manitoba, Canada; Dalhousie University, Canada

## Abstract

Adenosine, through activation of its A_1_ receptors, has neuroprotective effects during hypoxia and ischemia. Recently, using transgenic mice with neuronal expression of human equilibrative nucleoside transporter 1 (hENT1), we reported that nucleoside transporter-mediated release of adenosine from neurons was not a key mechanism facilitating the actions of adenosine at A_1_ receptors during hypoxia/ischemia. The present study was performed to test the importance of CD73 (ecto-5′-nucleotidase) for basal and hypoxic/ischemic adenosine production. Hippocampal slice electrophysiology was performed with CD73^+/+^ and CD73^−/−^ mice. Adenosine and ATP had similar inhibitory effects in both genotypes, with IC_50_ values of approximately 25 µM. In contrast, ATP was a less potent inhibitor (IC_50_ = 100 µM) in slices from mice expressing hENT1 in neurons. The inhibitory effects of ATP in CD73^+/+^ and CD73^−/−^ slices were blocked by the adenosine A_1_ receptor antagonist 8-cyclopentyl-1,3-dipropylxanthine (DPCPX) and were enhanced by the nucleoside transport inhibitor S-(4-nitrobenzyl)-6-thioinosine (NBTI), consistent with effects that are mediated by adenosine after metabolism of ATP. AMP showed a similar inhibitory effect to ATP and adenosine, indicating that the response to ATP was not mediated by P2 receptors. In comparing CD73^−/−^ and CD73^+/+^ slices, hypoxia and oxygen-glucose deprivation produced similar depression of synaptic transmission in both genotypes. An inhibitor of tissue non-specific alkaline phosphatase (TNAP) was found to attenuate the inhibitory effects of AMP and ATP, increase basal synaptic activity and reduce responses to oxygen-glucose deprivation selectively in slices from CD73^−/−^ mice. These results do not support an important role for CD73 in the formation of adenosine in the CA1 area of the hippocampus during basal, hypoxic or ischemic conditions, but instead point to TNAP as a potential source of extracellular adenosine when CD73 is absent.

## Introduction

ATP and adenosine inhibit synaptic transmission in electrically stimulated hippocampal slices [1]. The inhibitory effect of adenosine is mediated by adenosine A_1_ receptors, as determined through the use of selective antagonists and A_1_ receptor knockout (^−/−^) mice [1]. ATP appears to also act through A_1_ receptors as its inhibitory effects are blocked by A_1_ selective antagonists, but not by purinergic P2 receptor antagonists [2]. Furthermore, the inhibitory effects of ATP are not observed in A_1_ receptor^−/−^ mice [1]. Since ATP does not activate A_1_ receptors directly, this indicates that ATP is rapidly metabolized to adenosine and its inhibitory effects are actually mediated by adenosine [3].

Extracellular ATP can be metabolized to adenosine by a combination of enzymes. Ecto-nucleoside triphosphate diphosphohydrolases (E-NTPDases; ecto-apyrases; CD39), ecto-nucleotide pyrophosphatase/phosphodiesterases (E-NPPs) and alkaline phosphatases metabolize ATP and ADP to AMP, whereas alkaline phosphatases and CD73 (ecto-5′-nucleotidase; EC 3.1.3.5) can metabolize AMP to adenosine [4]. However, inhibitors of these enzymes have modest efficacy to decrease the effects of ATP or AMP and can have inhibitory effects of their own [3], [5]–[7]. It has been difficult to demonstrate conclusively that the inhibitory effects of exogenous adenine nucleotides result from their metabolism extracellularly to adenosine, in part, because their slow metabolism of variable efficacy is in contrast to their rapid inhibition of synaptic activity [2], [7].

Recently, we developed transgenic (Tg) mice that express human equilibrative nucleoside transporter 1 (hENT1) under the control of a neuron-specific promoter [8]. Radioligand binding assays showed a 20-fold increase in ENT1 abundance in Tg hippocampal membranes, relative to membranes from wild type (Wt) mice [9]. Using hippocampal slice electrophysiology, we reported that the potency of applied adenosine was decreased in slices from hENT1 Tg mice, indicating that increased cellular uptake of adenosine led to decreased adenosine A_1_ receptor activation [9]. Furthermore, both hypoxic and oxygen-glucose deprivation conditions produced less inhibition of synaptic activity in slices from hENT1 Tg mice, relative to slices from Wt littermate controls [9]. From this, we concluded that hypoxic/ischemic conditions do not trigger equilibrative transporter-mediated release of adenosine *per se* from neurons, despite rapid decreases in neuronal ATP levels. Instead, we proposed that adenosine is released from another cell type or via another mechanism, or ATP (or another nucleotide) is released and metabolized extracellularly to adenosine during hypoxic/ischemic conditions [9].

**Figure 1:**
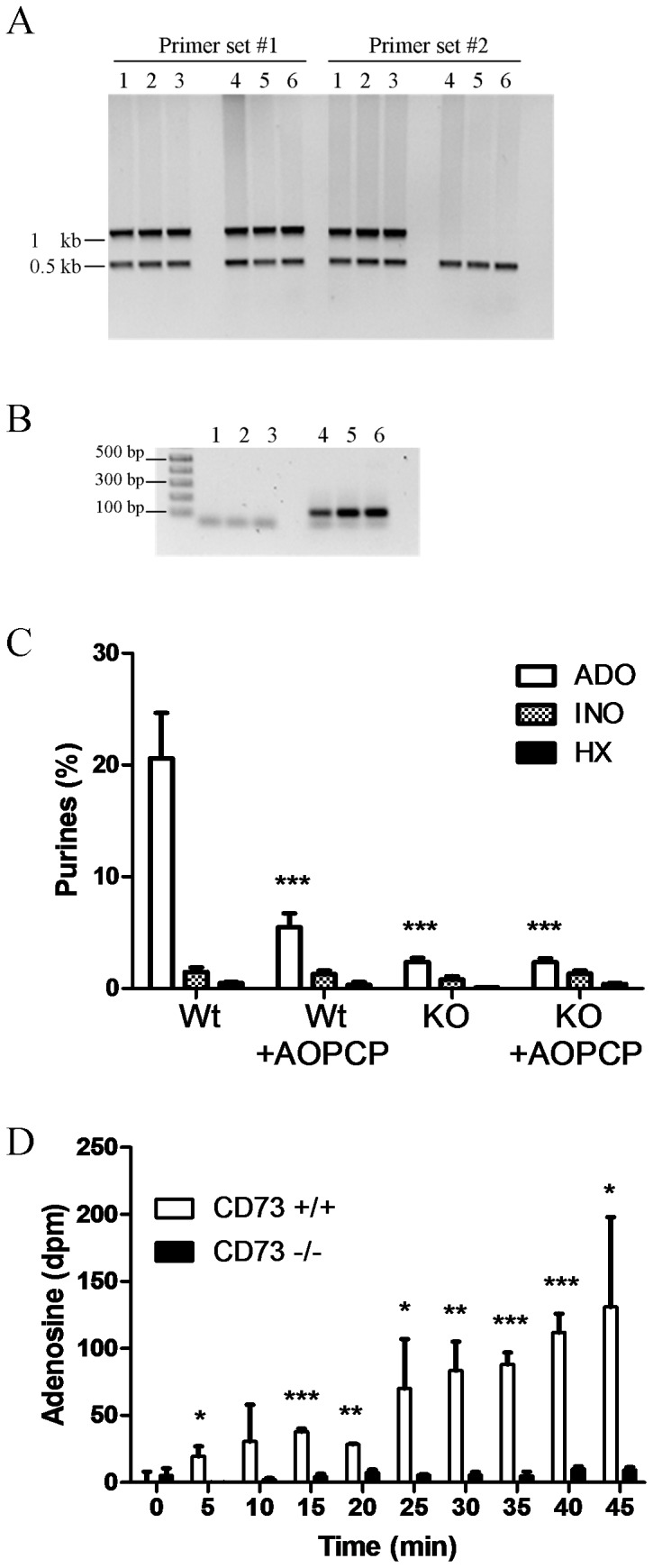
Loss of ecto-5′-nucleotidase activity in CD73^−/−^ mice. (a) Genomic DNA was isolated from three CD73^−/−^ (1, 2, 3) and three CD73^+/+^ (4, 5, 6) mice. Multiplex PCR was performed using primer set 1, to amplify exon 10–11, or primer set 2, to amplify from the region of mutated DNA, together with primers to mouse beta casein. While PCR amplified the beta casein internal control gene and the 3′end of CD73 in all samples, only samples from CD73^−/−^ mice produced a PCR product from the mutated region of the CD73 gene. (b) Total RNA was isolated from cerebral cortex from three CD73^−/−^ (1, 2, 3) and three CD73^+/+^ (4, 5, 6) mice. RNA was reverse transcribed and cDNA for CD73 exons 3–4 was amplified using primer set 3. A PCR product of the expected size was obtained with cDNA from CD73^+/+^ but not CD73^−/−^ mice. (c) Membrane preparations from cerebral cortex from CD73^+/+^ and CD73^−/−^ mice were incubated with ^14^C-AMP (100 µM) and metabolism to adenosine, inosine and hypoxanthine was measured. AOPCP (50 µM) was added to some samples to inhibit CD73 (ecto-5′-nucleotidase) activity. Data are pmol/µg protein expressed as a percentage of total ^14^C-AMP applied, from 3 assays performed in triplicate. ***p<0.001, one way ANOVA with Tukey's post-hoc tests. (d) Hippocampal slices from CD73^+/+^ and CD73^−/−^ mice were incubated with ^14^C-AMP (100 µM) and metabolism to adenosine, inosine and hypoxanthine was measured before (0–15 min) or during (15–45 min) electrical stimulation. Data are disintegrations per min from 2 CD73^+/+^ or 4 CD73^−/−^ slices (1 slice per animal). *p<0.05, **p<0.01, ***p<0.001, two-tailed *t*-tests.

To address these potential mechanisms, the present study was performed. As CD73 is a key enzyme for the extracellular formation of adenosine [4], we used CD73^+/+^ and CD73^−/−^ mice to test whether CD73 deficiency affects responses to adenosine, ATP, hypoxia or oxygen-glucose deprivation in hippocampal slice preparations. Previous studies have reported that both adenosine formation and adenosine receptor activity were reduced in CD73^−/−^ mice [10]–[13]. In addition, tissue-nonspecific alkaline phosphatase (TNAP) has been shown to metabolize extracellular ATP in cultured hippocampal neurons and regulate axonal growth [14]. Therefore, we also tested whether TNAP affects responses to ATP, AMP, hypoxia or oxygen-glucose deprivation through the use of the inhibitor 2,5-dimethoxy-N-(quinolin-3-yl)benzenesulfonamide (TNAP-I) [15].

**Figure 2:**
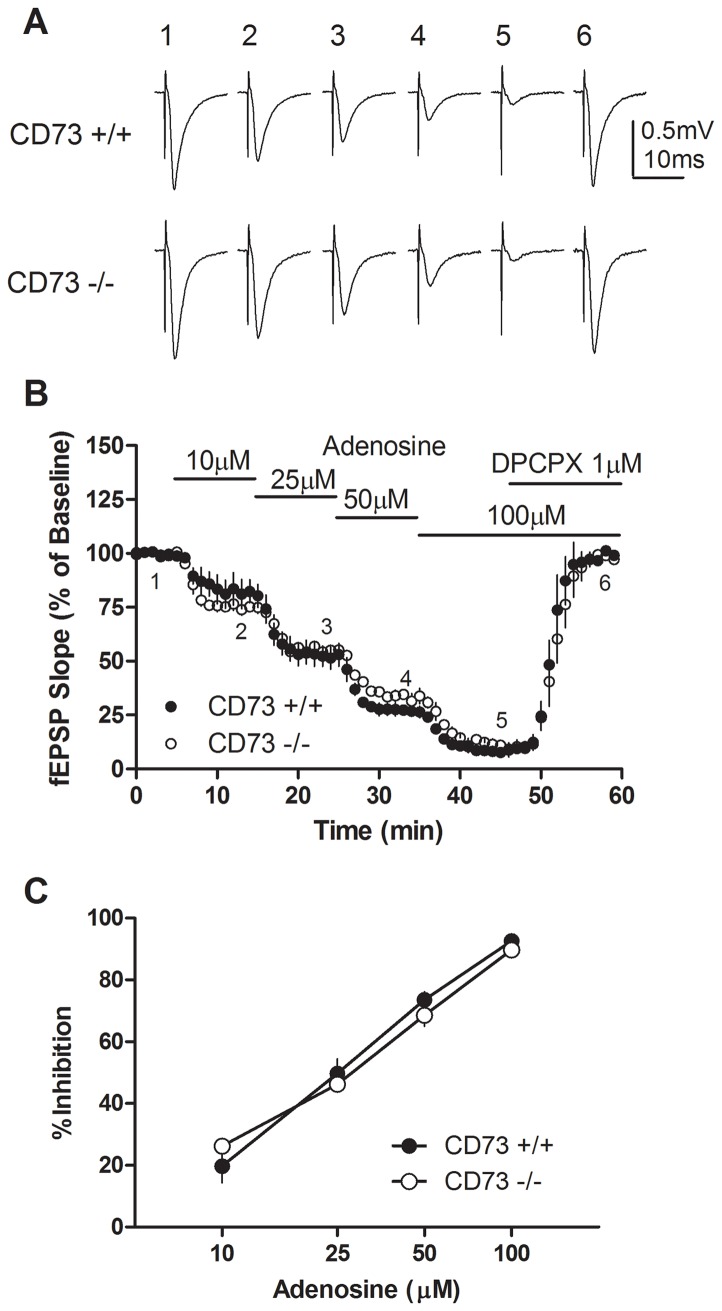
Inhibition by adenosine of fEPSP recordings in hippocampal slices from CD73^+/+^ and CD73^−/−^ mice. (a) Representative traces of fEPSPs; numbers 1–6 correspond to before and during superfusion of slices with adenosine (10, 25, 50 or 100 µM) or adenosine (100 µM) and DPCPX (1 µM) as indicated in (b). (b) The effect of adenosine on fEPSP slope in hippocampal slices from CD73^+/+^ and CD73^−/−^ mice. Horizontal lines represent the duration of superfusion with the indicated adenosine concentrations. (c) Maximum inhibition of fEPSPs obtained with each concentration of adenosine. *n* = 3 CD73^+/+^ and 3 CD73^−/−^.

## Materials and Methods

### Ethics statement

All procedures with animals were in accordance with guidelines set by the Canadian Council on Animal Care and approved by the University of Manitoba Animal Protocol Management and Review Committee.

### Mice

CD73^−/−^ mice were obtained from Dr. Linda Thompson [13]. Male CD73^−/−^ and wild type (CD73^+/+^) C57Bl6 mice were used at 8 weeks of age. In some experiments male mice expressing hENT1 under the control of neuron specific enolase promoter, and wild type littermates, were used at 8 weeks of age [8].

### PCR and reverse transcriptase PCR for CD73

Genomic DNA was extracted from tail snips using the Wizard® Genomic DNA Purification Kit (Promega Corporation), following the manufacturer's protocol. RNA was isolated from cortex or hippocampus samples using the TRIzol™ method (Invitrogen). The concentration of RNA was measured using a GeneQuant Pro spectrophotometer (Biochrom, Ltd.) and diluted to 1.5 µg/µL using ddH_2_O. Reverse transcription (RT) was performed by first treating 1.5 µg RNA with 1 U DNase (Invitrogen) in 20 mM Tris-HCl (pH 8.4), 2 mM MgCl_2_ and 50 mM KCl at room temperature for 15 min. Then, 500 ng olig(dT)_12–18_ primer and was added and heated to 65°C for 5 min. The final step consisted of the addition of Moloney-murine leukemia virus reverse transcriptase (200 U), dNTPs (0.5 mM), dithiothreitol (DTT; 10 mM), Tris-HCl (50 mM; pH 8.3), KCl (75 mM) and MgCl_2_ (3 mM) in a final volume of 60 µL, then incubated at 37°C for 1 h followed by 90°C for 5 min.

PCR was performed with genomic DNA or cDNA. Primer set 1 was 5′-GTGACCCTCCCAAGCTATCTG and 5′-GCTTCCCTGGTAATGACTTGC; these primers span exons 10 and 11 of CD73 and are predicted to amplify a sequence of 1.1 kb. Primer set 2 was 5′-AAGGAGGGGTGCATCTTGCTATTC and 5′-CCAGCTCATTCCTCCCACTCATG; these primers target intron 2 and the mutated sequence in exon 3 [13] and are predicted to amplify a sequence of 1 kb only in CD73^−/−^ mice. Primer set 3 was 5′-TCAGAAAGTTCGAGGTGTGGA and 5′-GTCCATCATCTGCGGTGACTA; these primers span exons 3 and 4 of CD73 and are predicted to amplify a sequence of 128 bp in wild type cDNA from cerebral cortex. Multiplex PCR was performed using CD73 and mouse beta casein gene specific primers, as described previously [8]. Ready To Go™ PCR beads (GE Healthcare) and 500 nM of primer mix (forward and reverse) were used to amplify 1 µL of DNA or cDNA. A PTC-100™ Programmable Thermal Controller (MJ Research, Inc.) was used for the amplification. The ‘slowdown PCR’ program [16] was used with cDNA because it had been found previously to reduce primer-dimers and give cleaner results.

A 1% agarose gel, containing ethidium bromide for visualization, was run in Tris acetate-EDTA (TAE) buffer (40 mM Tris acetate and 1 mM EDTA) for 30 −90 min at 105 V and the PCR products were cut from the gel while being illuminated by a FBTI-88 Transilluminator (Fisher Scientific).

### Quantitative PCR for TNAP

For quantitative PCR reaction mixtures, with a final volume of 50 µl, consisted of 1 µl reverse transcribed cDNA, 0.5 µM primers, 2.5 mM MgCl_2_, 0.2 mM dNTPs, 0.1× SYBR Green I, 0.25 U Platinum Taq polymerase and10 nM fluorescent calibration dye. The reactions consisted of 4 min at 95°C then 50 cycles of 95°C for 15 sec, 64°C for 15 sec, 72°C for 30 sec. This was immediately followed by a melt curve analysis consisting of 95°C for 1 min, 64°C for 1 min and 64°C for 10 sec, with the latter step increasing in temperature by 0.5°C per cycle for 63 cycles. Melt curve analysis confirmed the presence of a single PCR product in each reaction. A Bio-Rad iCycler iQ Real-Time PCR Detection System was used for these experiments.

Primer sequences for β-actin were 5′- CATGGCTGGGGTGTTGAAGGTTCT and 5′- CGAGCCCCAGAGCAAGAGAGGT and for TNAP were 5′- ACGGACATCATGAGGGTAAGG and 5′- CGTGGGAATGATCAGCAGTAA. Expected product sizes were 188 bp and 132 bp for β-actin and TNAP, respectively. For both primer pairs, standard curves were linear over a range of 5–6 log units and reaction efficiencies were between 90–110%.

### Ecto-5′-nucleotidase assays

Cell membranes were isolated from cortex obtained from CD73^+/+^ and CD73^−/−^ mice using previously published methods [8]. Briefly, cortex from each mouse was homogenized in 10 volumes of ice-cold 0.32 M sucrose then centrifuged at 1000× *g* for 10 min at 4°C. The pellet was washed twice and pooled supernatants were centrifuged at 20,000× *g* for 30 min at 4°C. The resulting pellets were resuspended in HEPES buffer composed of 110 mM NaCl, 25 mM glucose, 68.3 mM sucrose, 5.3 mM KCl, 1.8 mM CaCl_2_, 1.0 mM MgSO_4_ and 20 mM HEPES, pH 7.4. Protein concentrations were determined with a Bio-Rad dye binding assay.

Ecto-5′-nucleotidase assays were performed in triplicate in an assay volume of 0.3 ml with 100 µM ^14^C-AMP and 40 µg membrane protein per assay for 15 min at room temperature. α,β-methylene ADP (AOPCP; 50 µM) was used to inhibit ecto-5′-nucleotidase and TNAP-I was used to inhibit TNAP (10 µM). Assays were terminated by centrifugation (14,000× *g*; 2 min). ^14^C-purines (AMP, adenosine, inosine and hypoxanthine) were separated by thin layer chromatography, using *n*-butanol, ethyl acetate, methanol and ammonium hydroxide in a ratio of 7∶4∶3∶4 vol/vol, then quantified by scintillation spectroscopy [17], [18]. The amount of adenosine, inosine and hypoxanthine produced was expressed as a percentage of the AMP added.

Ecto-5′-nucleotidase assays were also performed with hippocampal slices, prepared as described below. Hippocampal slices were kept at 32.5°C with 1 ml of artificial cerebrospinal fluid (aCSF) containing 10 µM [^14^C]AMP. Samples (50 µl) were taken at 5 min intervals for 45 min and analyzed for [^14^C]adenosine content by thin layer chromatography. After the first 4 sample collections, slices were electrically stimulated as described below for the remaining 30 min.

### Hippocampal slice preparation

Mice were anaesthetized with isoflurane and decapitated. Each brain was rapidly removed in ice-cold oxygenated (95% O_2_/5% CO_2_) aCSF of the following composition (mM): 124 NaCl, 3.0 KCl, 1.2 MgCl_2_, 2.0 CaCl_2_, 1.25 KH_2_PO_4_, 26 NaHCO_3_, 10 D-glucose, pH 7.4, adjusted to 285–290 mOsm. Hippocampus was removed and 350 µm slices were cut using a McIlwain tissue chopper (Stoelting Co, Wood Dale, IL). The slices were then allowed to recover in a holding chamber for at least 1 hr at 32.5°C prior to electrophysiological recording or ecto-5′-nucleotidase assays.

### Extracellular recording

Individual slices were transferred to a submerged recording chamber (Harvard Apparatus, Holliston, MA) and continuously superfused with oxygenated aCSF at a flow rate of 1.5 ml/min (32.5°C). Each slice was placed on a suspended nylon net to allow perfusion and oxygenation of the slice from above and below. Synaptic responses were evoked by stimulation of the Schaffer collateral/commissural pathway with a concentric bipolar stimulating electrode with 0.1 millisecond pulse width at 30 second intervals. Extracellular field excitatory postsynaptic potentials (fEPSPs) were recorded in stratum radiatum of CA1 hippocampus using glass microelectrodes (1–2 Ω) filled with aCSF. Input/output curves were obtained by gradual increases in stimulus voltage at the beginning of each experiment, when a stable baseline of fEPSP response was reached. The baseline recording was established at 40–50% maximal fEPSP response. At least 15 to 20 min of stable baseline was obtained in each experiment. Data were collected using an Axopatch 1D amplifier and analyzed using pCLAMP 10 software (Molecular Devices, Sunnyvale, CA).

### Application of hypoxia or oxygen-glucose deprivation

*In vitro* hypoxia, 10 min, was induced by superfusing slices with aCSF gassed with 95% N_2_/5% CO_2_. *In vitro* oxygen-glucose deprivation, 3 min, was induced by superfusing slices with aCSF prepared with sucrose instead of glucose and gassed with 95% N_2_/5% CO_2_.

### Drugs

ATP, adenosine, AOPCP, S-(4-nitrobenzyl)-6-thioinosine (NBTI), 8-cyclopentyl-1,3-dipropylxanthine (DPCPX), dimethylsulfoxide (DMSO) were purchased from Sigma-Aldrich Canada Ltd (Oakville ON). TNAP-I was purchased from Calbiochem (Mississauga, ON). Adenosine and ATP were prepared in distilled water then diluted to 10–100 µM in aCSF. A spectrophotometer was used to ensure the accuracy of final concentrations using the extinction coefficients at λ 259 of 15.1 mm^−1^·cm^−1^ and 15.4 mm^−1^·cm^−1^ for adenosine and ATP, respectively. NBTI, DPCPX and TNAP-I were dissolved in DMSO, then diluted in aCSF to obtain DMSO concentrations of 0.01% (NBTI, DPCPX) or 0.1% (TNAP-I).

### Statistical analysis

All numerical data are expressed as means ± SEM. Data were tested for statistical significance with two-tailed Student's *t*-test or one way ANOVA with Tukey's post hoc tests. A value of *p*<0.05 was considered statistically significant.

## Results

### Characterization of CD73^−/−^ mouse model

PCR, reverse transcriptase PCR and ecto-5′-nucleotidase assays were performed to verify the absence of CD73 in CD73^−/−^ mice and its presence in CD73^+/+^ mouse samples. Primer set 1, which targets exons 10 and 11, amplified a 1.1 kb sequence from genomic DNA of both CD73^+/+^ and CD73^−/−^ mice (Fig. 1A). Primer set 2, which targets the region of the gene that was mutated to produce the knock out phenotype, amplified a 1 kb sequence from genomic DNA isolated from CD73^−/−^ mice. As expected these primers did not produce a PCR product using CD73^+/+^ DNA (Fig. 1A). Primer set 3, which targets exon 3–4, the region disrupted in the genome of the CD73^−/−^ mice, amplified a 128 bp sequence of cDNA from CD73^+/+^ but not CD73^−/−^ cortex (Fig. 1B) and hippocampus (data not shown). Thus, the PCR and reverse transcriptase PCR results confirm that the CD73 gene and its expression are disrupted in CD73^−/−^ mice.

Ecto-5′-nucleotidase assays were performed with cell membranes isolated from cerebral cortex. Approximately 21±4% (221±7.5 pmol/µg protein) of the ^14^C-AMP was hydrolyzed to adenosine in assays using samples from CD73^+/+^ mice. AOPCP (50 µM) significantly inhibited adenosine formation, with 5±1% (62±1.3 pmol/µg protein) of the AMP metabolized to adenosine. In contrast, in parallel assays using samples from CD73^−/−^ mice, less than 3% (24±1.3 pmol/µg protein) of AMP was metabolized to adenosine and AOPCP had no effect (26±0.5 pmol/µg protein) (Fig. 1C). Inosine and hypoxanthine were also quantified, but their levels were less than 3% of AMP both with CD73^−/−^ and CD73^+/+^ samples and were unaffected by AOPCP.

Conversion of [^14^C]AMP to [^14^C]adenosine was also examined in hippocampal slices. Significantly greater quantities of [^14^C]adenosine were detected in superfusate of CD73^+/+^ slices, relative to CD73^−/−^ slices (Fig. 1D), both before (0–15 min) and during (15–45 min) electrical stimulation.

### Hippocampal slices from CD73^+/+^ and CD73^−/−^ mice show similar responses to adenosine and ATP

Adenosine produced a concentration dependent decrease in synaptic activity, with an IC_50_ value of approximately 25 µM and almost complete inhibition was observed with 100 µM adenosine (Fig. 2A–B). This inhibitory effect of adenosine was mediated by adenosine A_1_ receptors, since the A_1_ selective antagonist DPCPX restored synaptic activity to control levels (Fig. 2A). As expected, no significant differences in the effects of adenosine were observed between hippocampal slices obtained from CD73^+/+^ or CD73^−/−^ mice.

**Figure 3:**
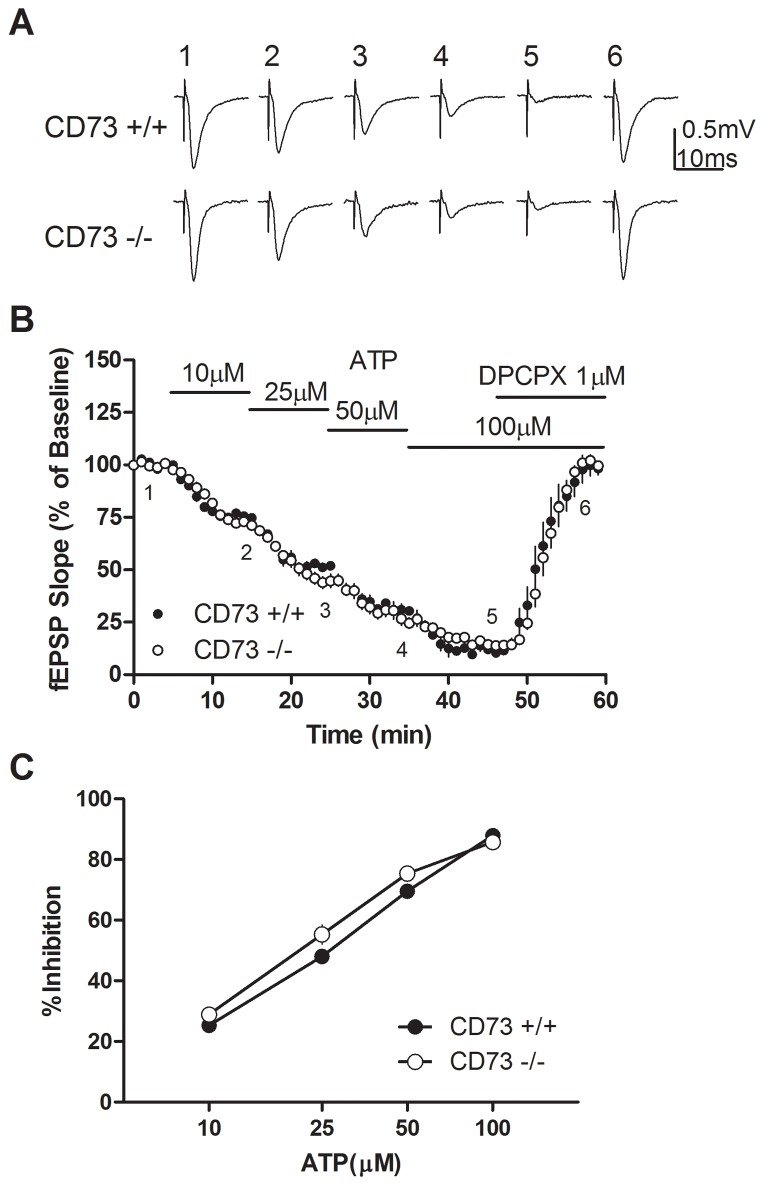
Inhibition by ATP of fEPSP recordings in hippocampal slices from CD73^+/+^ and CD73^−/−^ mice. (a) Representative traces of fEPSPs; numbers 1–6 correspond to before and during superfusion of slices with ATP (10, 25, 50 or 100 µM) or ATP (100 µM) and DPCPX (1 µM) as indicated in (b). (b) The effect of ATP on fEPSP slope in hippocampal slices from CD73^+/+^ and CD73^−/−^ mice. Horizontal lines represent the duration of superfusion with the indicated ATP concentrations. (c) Maximum inhibition of fEPSPs obtained with each concentration of ATP. *n* = 4 CD73^+/+^ and 5 CD73^−/−^.

The absence of ecto-5′-nucleotidase activity is predicted to reduce or abolish the effects of ATP that are sensitive to inhibition by DPCPX. However, ATP produced a concentration dependent decrease in synaptic activity in both CD73^+/+^ or CD73^−/−^ hippocampal slices (Fig. 3A). The IC_50_ value for ATP was approximately 25 µM, and 100 µM produced almost complete cessation of synaptic activity (Fig. 3B). As with adenosine, DPCPX blocked the effects of ATP. While the overall effects of ATP were very similar to adenosine, one noticeable difference was that there was a slower response to each individual concentration of ATP (compare Fig. 2A, 3A), indicating that responses to ATP were slower to equilibrate. There was a trend for a greater inhibitory effect of ATP in CD73^−/−^ slices compared to CD73^+/+^ slices, which was statistically significant (*p*<0.05) at 25 µM ATP.

### In contrast to CD73^−/−^ mice, hENT1 transgenic mice show reduced responses to ATP

**Figure 4:**
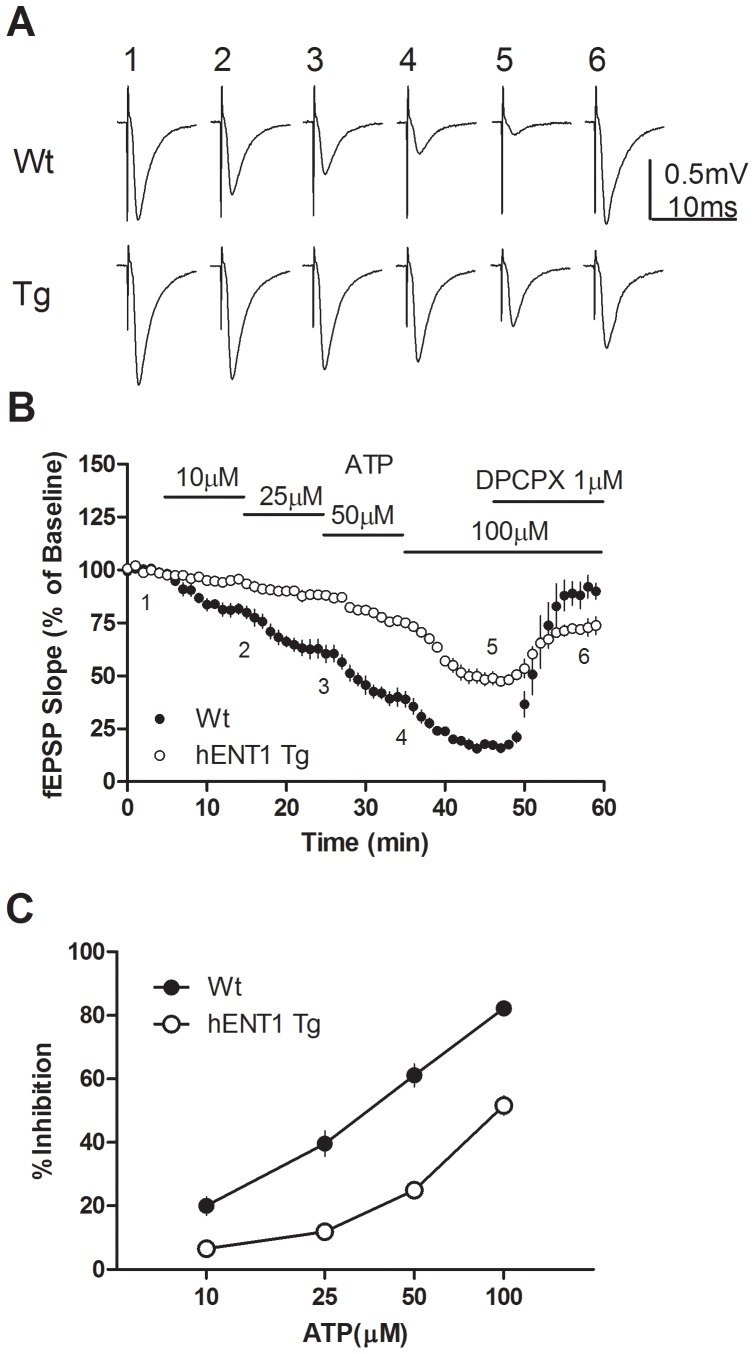
Inhibition by ATP of fEPSP recordings in hippocampal slices from hENT1 Tg and Wt mice. (a) Representative traces of fEPSPs; numbers 1–6 correspond to before and during superfusion of slices with ATP (10, 25, 50 or 100 µM) or ATP (100 µM) and DPCPX (1 µM) as indicated in (b). (b) The effect of ATP on fEPSP slope in hippocampal slices from hENT1 Tg and Wt littermate mice. Horizontal lines represent the duration of superfusion with the indicated ATP concentrations. (c) Maximum inhibition of fEPSPs obtained with each concentration of ATP. *n* = 4 hENT1 Tg and 4 Wt.

Previously, we reported that exogenous adenosine was a more potent inhibitor of synaptic activity in wild type, compared to hENT1 transgenic, hippocampal slices [9]. To test whether enhanced neuronal uptake of adenosine affects hippocampal responses to ATP, we used slices from hENT1 transgenic and wild type littermates. Similar to results with adenosine in hENT1 transgenic mice, but in contrast to results with CD73^+/+^ and CD73^−/−^ slices, ATP had significantly reduced potency for inhibiting synaptic activity in hENT1 transgenic slices, relative to wild type slices (Fig. 4).

### Basal adenosine levels are similar in hippocampal slices from CD73^+/+^ and CD73^−/−^ mice

**Figure 5:**
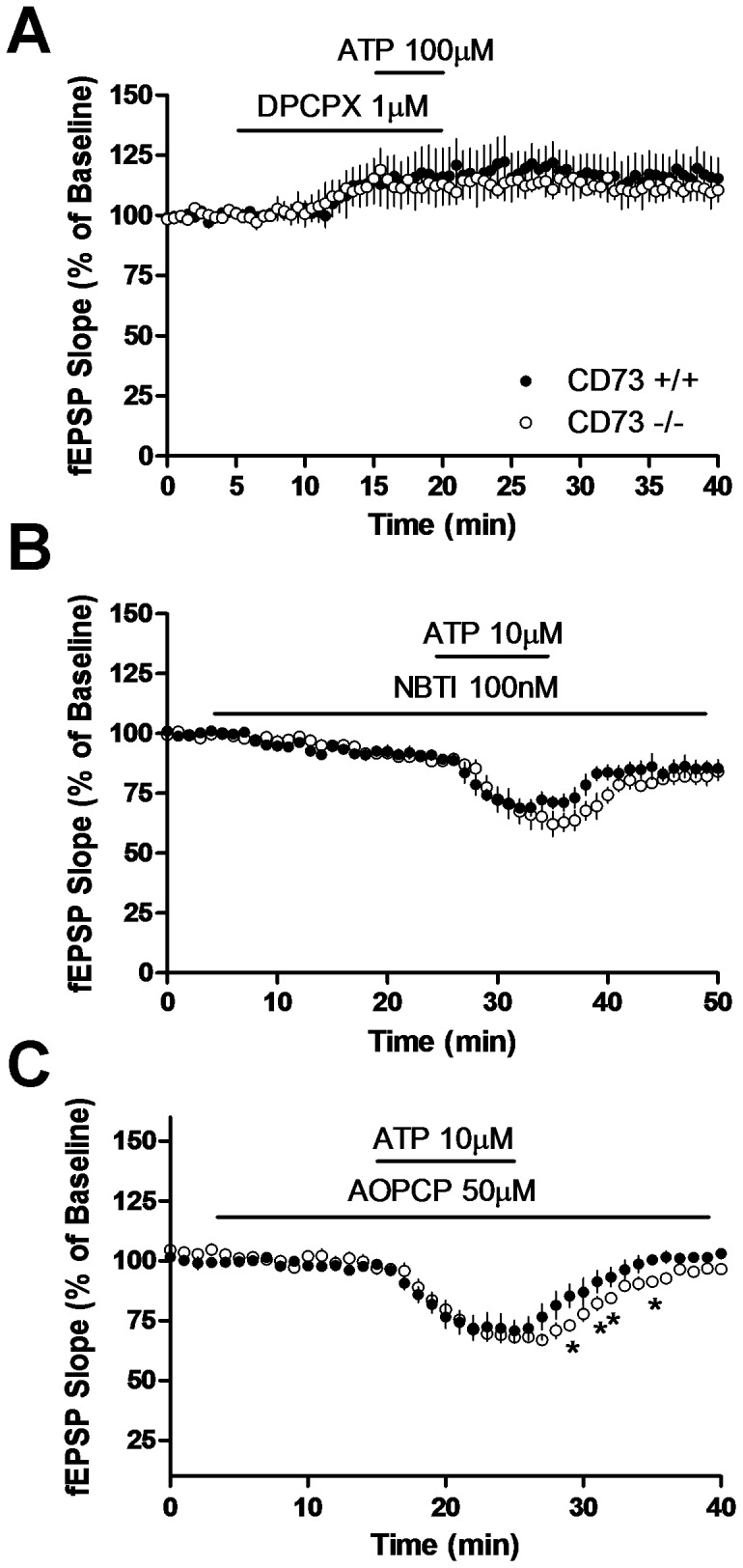
DPCPX and NBTI, but not AOPCP, affect inhibition of fEPSPs by ATP. Hippocampal slices from CD73^+/+^ and CD73^−/−^ mice were superfused with (a) 1 µM DPCPX, (b) 100 nM NBTI or (c) 50 µM AOPCP as indicated by the horizontal lines. After 10 min (a, c) or 20 (b) min, 100 µM ATP was applied as indicated by second horizontal line. *n* = 3–4 CD73^+/+^ and 3–4 CD73^−/−^. *p<0.05; two tailed *t*-test.

DPCPX tended to increase synaptic activity in hippocampal slices from both CD73^+/+^ and CD73^−/−^ mice (Fig. 5A), although a high degree of variability between samples was evident. After 10 min treatment with DPCPX, fEPSP slope values were 114±9% and 115±8% in CD73^+/+^ and CD73^−/−^, respectively. DPCPX prevented the inhibitory effects of ATP (Fig. 5A); after 5 min treatment with ATP in the continued presence of DPCPX, fEPSP slope remained at 116±12% and 113±7% in CD73^+/+^ and CD73^−/−^, respectively.

NBTI, a selective inhibitor of ENT1, produced a slowly developing inhibition of synaptic activity (Fig. 5B). After 20 min treatment with NBTI, fEPSP slope was decreased to 89±1% in both CD73^+/+^ and CD73^−/−^ slices. In the presence of NBTI, treatment for 10 min with ATP produced a further decrease in synaptic activity, to 71±3% and 62±5% in CD73^+/+^ and CD73^−/−^, respectively.

AOPCP, an inhibitor of ecto-5′-nucleotidase, had no effect on synaptic activity in slices from CD73^+/+^ and CD73^−/−^ (Fig. 5C). In the presence of AOPCP, treatment for 10 min with ATP (10 µM), produced a decrease in fEPSP values to 69±4% and 68±3% in CD73^+/+^ and CD73^−/−^, respectively. Field EPSP values returned to 101±1 and 98±1% in CD73^+/+^ and CD73^−/−^, respectively, upon washout of ATP but recovery was significantly slower in CD73^−/−^ slices (*p*<0.05).

### Hippocampal slices from CD73^+/+^ and CD73^−/−^ mice show similar responses to hypoxia and to oxygen-glucose deprivation

**Figure 6:**
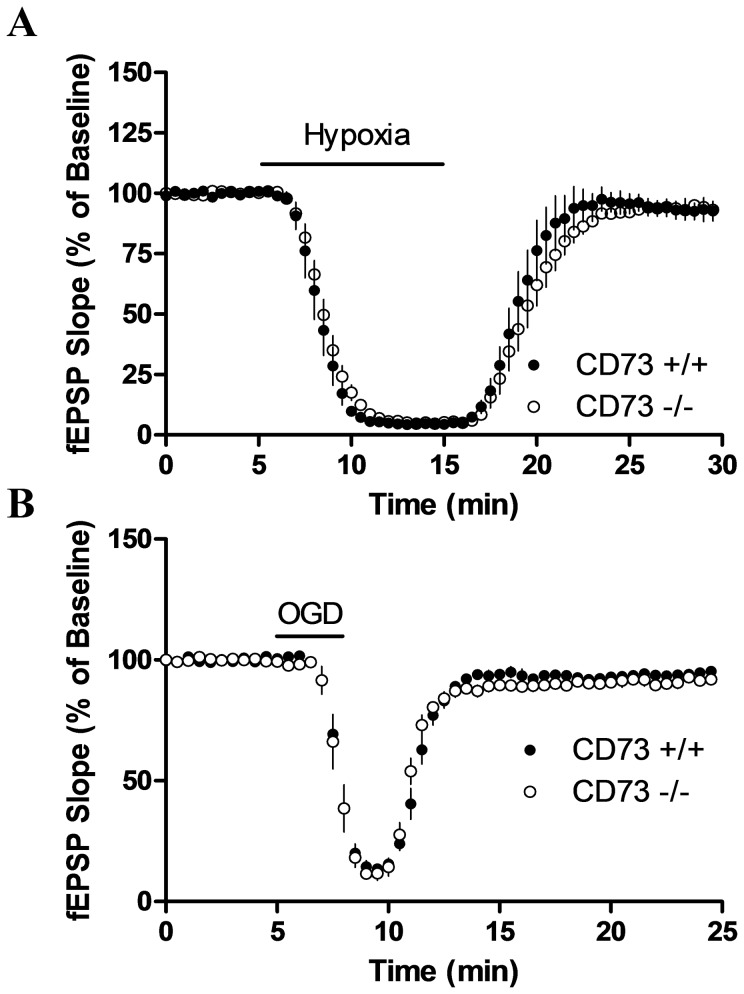
Effect of hypoxia or oxygen-glucose deprivation (OGD) on fEPSP recordings. (a) Slices were superfused with hypoxic aCSF for 10 min as indicated by the horizontal bar. *n* = 7 CD73^+/+^ or 9 CD73^−/−^ mice. (b) Slices were superfused with hypoxic glucose-free aCSF for 3 min as indicated by the horizontal bar. *n* = 5 CD73^+/+^ or 4 CD73^−/−^ mice. There were no significant differences between genotypes.

Previous studies have demonstrated hypoxia produces synaptic inhibition that is caused, in part, by adenosine acting at A_1_ receptors [19], [20]. Our previous study showed that this adenosine is not released via equilibrative transporters from neurons [9], but the study did not examine whether it was due to metabolism of extracellular ATP to adenosine. Using hippocampal slices from CD73^+/+^ and CD73^−/−^ mice, hypoxia (10 min) produced a complete inhibition of synaptic activity in both (Fig. 6A). There was a trend towards a slower recovery with CD73^−/−^ slices, but this was not statistically significant. The recovery of synaptic activity after reoxygenation was similar, reaching 93±2% and 93±5% in CD73^+/+^ and CD73^−/−^ slices, respectively.

Hippocampal slices from CD73^+/+^ and CD73^−/−^ mice were also exposed to oxygen-glucose deprivation (3 min); a pronounced decrease in synaptic activity was observed and no differences between CD73^+/+^ and CD73^−/−^ in inhibitory response or recovery were detected (Fig. 6B).

### TNAP is an important source of adenosine in CD73^−/−^ but not CD73^+/+^ mice and contributes to adenosine production during oxygen-glucose deprivation

**Figure 7:**
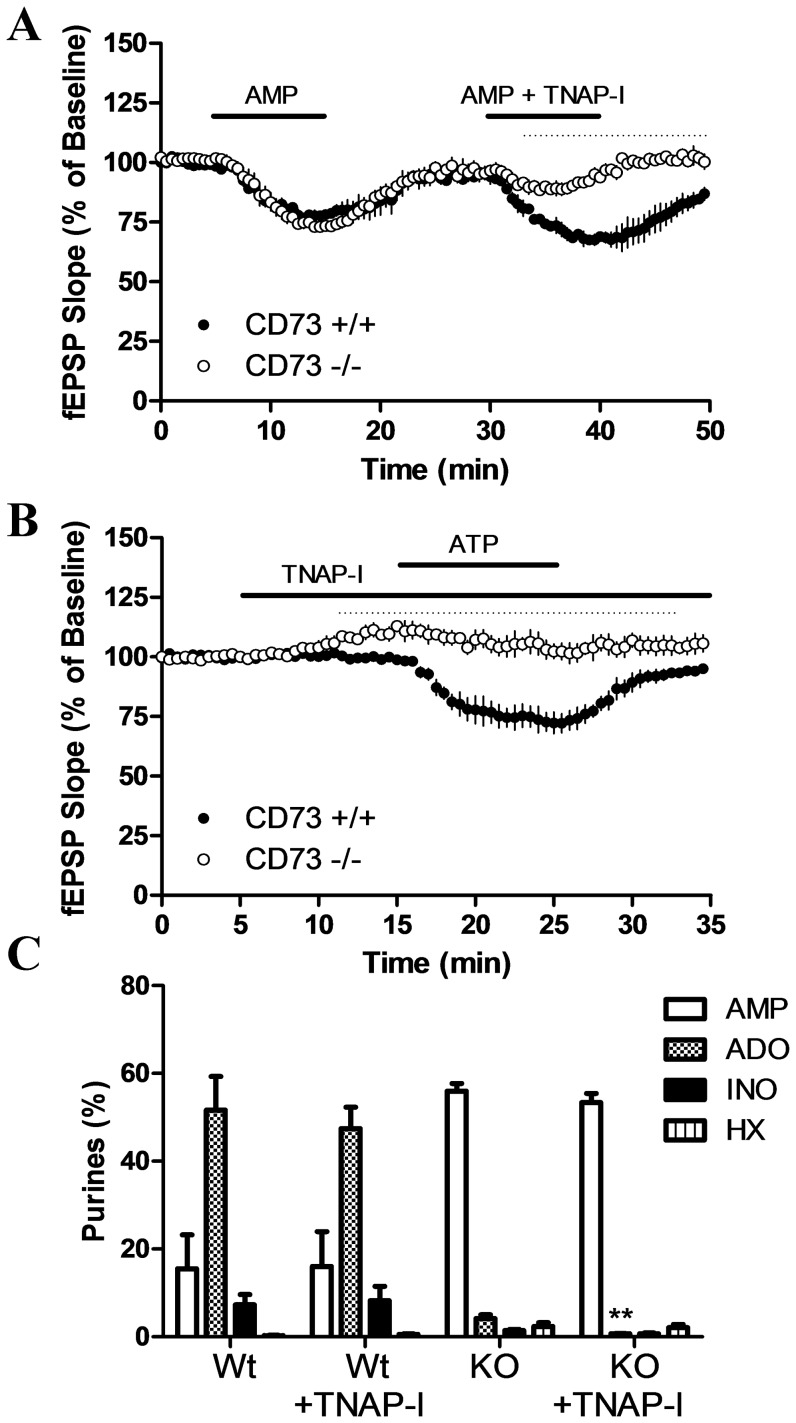
Inhibition of fEPSPs by AMP or ATP is reduced by TNAP-I selectively in CD73^−/−^ slices. (a) Hippocampal slices from CD73^+/+^ and CD73^−/−^ mice were superfused with 10 µM AMP as indicated by the horizontal bar. After 15 min recovery, slices were superfused with 10 µM AMP plus 10 µM TNAP-I as indicated by the second horizontal bar. *n* = 3 CD73^+/+^ and 3 CD73^−/−^. Significant differences between genotypes are indicated by dashed lines; *p*<0.05; unpaired *t*-tests. (b) Hippocampal slices from CD73^+/+^ and CD73^−/−^ mice were superfused with 10 µM TNAP-I and, after 10 min, with 10 µM ATP as indicated by the horizontal bars. *n* = 3 CD73^+/+^ and 3 CD73^−/−^. Significant differences between genotypes are indicated by dashed lines; *p*<0.05; unpaired *t*-tests. (c) Inhibition of metabolism of [^14^C]AMP to [^14^C]adenosine, [^14^C]inosine and [^14^C]hypoxanthine by TNAP-I was determined using membrane preparations from cerebral cortices. *n* = 4 CD73^+/+^ and 4 CD73^−/−^. *p<0.05, relative to absence of TNAP-I; unpaired *t*-test. Ado, adenosine; Ino, inosine; HX, hypoxanthine.

Similar to adenosine and ATP, AMP (10 µM) inhibited synaptic transmission in hippocampal slices from both CD73^+/+^ and CD73^−/−^ mice; after 10 min, fEPSP values were 78±2 and 73±1, respectively (Fig. 7A). For CD73^−/−^, but not CD73^+/+^, slices, this inhibition was attenuated by the application of TNAP-I (10 µM); after 10 min, fEPSP values were 97±1 and 68±3, respectively (Fig. 7A). TNAP-I produced a significant increase in fEPSPs in CD73^−/−^ hippocampal slices, consistent with a decrease in basal adenosine production; after 10 min fEPSPs were 113±2 and 99±1 for CD73^−/−^ and CD73^+/+^, respectively (*p*<0.05). Selectively in CD73^−/−^ slices, pre-treatment with TNAP-I abolished the inhibitory effects of 10 µM ATP (Fig. 7B).

Nucleotidase assays were performed to test the effect of TNAP-I. As shown in Fig. 7C, no effect of TNAP-I was observed in CD73^+/+^ cortical membranes, but a significant inhibition of adenosine production, from 3±0.2 to 0.7±0.1 pmol/µg protein, was observed in CD73^−/−^ samples.

**Figure 8:**
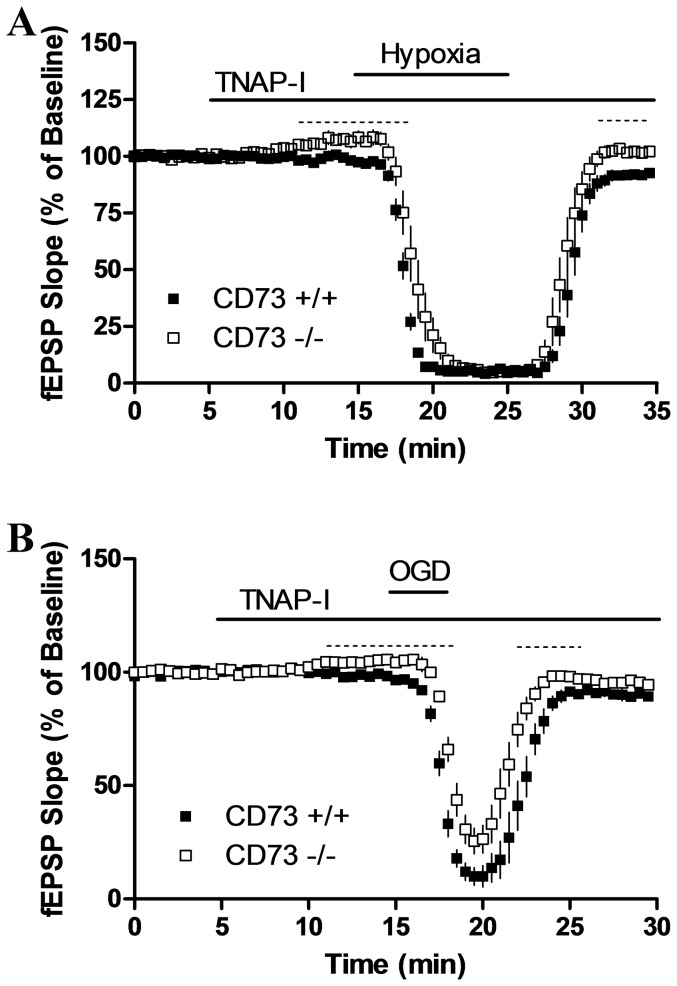
TNAP-I reduces oxygen-glucose deprivation (OGD)-induced decreases in fEPSP recordings from CD73^−/−^ mice. Slices were superfused with TNAP-I as indicated by the horizontal bar and were superfused with (a) hypoxic aCSF for 10 min or (b) hypoxic glucose-free aCSF for 3 min as indicated by the second horizontal bar. *n* = 6 CD73^+/+^ or 7 CD73^−/−^ mice (a) or from *n* = 5 CD73^+/+^ or 8 CD73^−/−^ mice (b). Significant differences between genotypes are indicated by dashed lines. *p*<0.05; unpaired *t*-tests.

To test the contribution of TNAP to inhibition of fEPSPs by hypoxia or oxygen-glucose deprivation, slices were superfused with TNAP-I prior to, during and following hypoxia (10 min) or oxygen-glucose deprivation (3 min) (Fig. 8A–B). In slices from CD73^−/−^ mice, TNAP-I produced an increase in basal synaptic transmission and decreased synaptic inhibition produced by transient oxygen-glucose deprivation (Fig. 8B) but not hypoxia (Fig. 8A).

### TNAP expression is unchanged in CD73 −/− mice

Since TNAP-I selectively enhanced synaptic transmission in slices from CD73^−/−^ and not CD73^+/+^ mice, RT-PCR and quantitative PCR were performed to examine for a genotype difference in expression of TNAP. RT-PCR indicated that TNAP was similarly expressed in hippocampus from CD73^+/+^ and CD73^−/−^ mice (data not shown). PCR data from CD73^+/+^ and CD73^−/−^ cortex cDNA were normalized to their respective β-actin values and expressed relative to CD73^+/+^ TNAP gene expression using the 2^−ΔΔCT^ method [21]. No significant difference in TNAP expression was detected: means and 95% confidence intervals were 1.00 (0.70–1.42) and 0.76 (0.52–1.13) for CD73^+/+^ and CD73^−/−^, respectively.

## Discussion

For the present study, we used CD73^−/−^ mice and tested the hypothesis that CD73 is required for basal and hypoxic/ischemic adenosine production in the CA1 region of the hippocampus. The main findings were that (1) CD73 metabolizes AMP to adenosine in cortical membranes and hippocampal slices; (2) CD73 is not required for the inhibitory effects of ATP in hippocampal slices; and (3) CD73 is not required for the inhibitory effects of adenosine observed in hypoxia or OGD. These data indicate the presence of another enzyme involved in the metabolism of extracellular ATP to adenosine. We show that TNAP-I inhibits responses to ATP and AMP selectively in CD73^−/−^ hippocampal slices but it has only a modest effect to attenuate synaptic inhibition in OGD. Therefore, the effects of exogenous ATP and endogenous adenosine are largely independent of both CD73 and TNAP in wild type mice.

We recently reported that transgenic mice expressing bidirectionalhENT1 in neurons showed decreased adenosine A_1_ receptor-mediated effects during basal conditions as well as during hypoxia and oxygen-glucose deprivation [9]. From these data we concluded that neuronal ENTs were more important for adenosine uptake than release and suggested that ATP released as a gliotransmitter from astrocytes may be an important source of adenosine during basal conditions as well as during conditions of hypoxia and oxygen-glucose deprivation. To follow up on these findings, we performed the experiments described here, with CD73^−/−^ mice. Our results do *not* provide support for an important role of CD73 in metabolizing extracellular ATP to adenosine during basal or hypoxic/ischemic conditions, but do point to TNAP as a potential source of extracellular adenosine when CD73 is absent.

Our electrophysiology data could suggest that ecto-5′-nucleotidase activity was still present in CD73^−/−^ mice. We confirmed the absence of CD73 using both PCR and reverse transcriptase PCR. In addition, we performed nucleotidase assays and found that CD73^+/+^ mouse brain samples clearly exhibited this activity, and at least 90% of this activity was lost in samples from CD73^−/−^ mice. Furthermore, AOPCP inhibited adenosine production from AMP only in samples from CD73^+/+^ mice. Metabolism of [^14^C]AMP to adenosine was also examined using hippocampal slices and electrical stimulation was applied to reproduce the conditions of slice electrophysiology. [^14^C]Adenosine was produced by slices from CD73^+/+^ mice but was very low in slices from CD73^−/−^ mice. Thus, CD73 is an important enzyme that produces adenosine from exogenously applied AMP, both in hippocampal slices and brain membrane preparations; however, it is possible that another enzyme is important in the membrane microdomains that contain A_1_ receptors.

The importance of CD73 for synaptic inhibition produced with ATP was examined. Although we hypothesized that the inhibitory effects of ATP, on synaptic transmission in hippocampal slices, would be abolished or significantly attenuated in slices from CD73^−/−^ mice, this was not the case. Not only was the potency of ATP similar between CD73^+/+^ mice and CD73^−/−^ mice, the potency was similar to the potency of adenosine, as shown here and as previously reported [1]–[3]. Previous studies have reported that the inhibitory effects of ATP are mediated by adenosine acting at A_1_ receptors, as they are blocked by A_1_ receptor antagonists and are not evident in adenosine A_1_ receptor^−/−^ mice [1]. ATP analogues such as ATPγS, β,γ-imido-ATP, and β,γ-methylene-ATP have similar potency for inhibiting fEPSPs as adenosine and ATP [1], [2], [5]. As the effects of these analogues are inhibited by adenosine deaminase and DPCPX, and potentiated by the nucleoside transport inhibitor dipyridamole, it was concluded that these ATP analogues require metabolism to adenosine and activation of A_1_ receptors for their effects [1], [2]. The availability of CD73^−/−^ mice allowed a new approach to evaluate the role of CD73 in regulating adenosine levels. However, instead of establishing the importance of CD73 for the inhibitory effects of ATP, our results support the opposite conclusion.

In the present study, DPCPX increased synaptic activity by blocking A_1_ receptor activity mediated by basal adenosine levels. DPCPX was effective in both preventing (Fig. 5) and blocking (Fig. 4) the inhibitory effects of ATP, as has been reported previously using theophylline [3] or 8-cyclopentyltheophylline [1]. NBTI, which decreased synaptic transmission by reducing cellular uptake of adenosine and enhancing basal adenosine levels, added to the inhibitory effect produced by ATP. AOPCP had no effect on synaptic transmission, indicating that CD73 was not involved in regulating basal adenosine levels. Attenuation of the effects of AMP by AOPCP has been reported previously by some [6] but not by others [3]. The effects of DPCPX, NBTI and AOPCP were similar in CD73^+/+^ and CD73^−/−^ slices, indicating that CD73 is not required for basal adenosine formation or adenosine formation from exogenous ATP.

Both hypoxia and oxygen-glucose deprivation decrease synaptic transmission, which is, in part, mediated by adenosine acting at A_1_ receptors [22], [23]. It has been reported that this adenosine is formed extracellularly, subsequent to release of ATP from glia [24]. However, from the data presented here, CD73 is not a key enzyme required for extracellular adenosine formation during these conditions.

One explanation for these data is that an enzyme other than CD73 is responsible for the very rapid generation of adenosine from released or exogenous ATP. TNAP is an enzyme that metabolizes extracellular ATP and reduces ATP-dependent inhibition of axonal growth in cultured neurons [14]. We examined the effect of TNAP-I, reported to inhibit TNAP with an inhibitory constant (K_i_) of 600 nM [15]. Interestingly, the inhibitory effects of AMP were significantly reduced by TNAP-I, but only in slices from CD73^−/−^ mice. Furthermore, synaptic inhibition produced by oxygen-glucose deprivation was attenuated, but only in slices from CD73^−/−^ mice. These data indicate that TNAP is a significant contributor to adenosine production when CD73 is absent. From quantitative PCR of cDNA samples, we found similar expression of TNAP in CD73^+/+^ and CD73^−/−^ brain samples, yet formation of [^14^C]adenosine from [^14^C]AMP was significantly inhibited by TNAP-I in CD73^−/−^ but not CD73^+/+^ samples. Therefore, in the absence of CD73, TNAP had a measurable effect on adenosine production, but this was not evident in samples that contain CD73, likely because the abundant CD73 activity masked the small contribution of TNAP. TNAP-I is a selective inhibitor of TNAP [15] and TNAP expression was similar in CD73^+/+^ and CD73^−/−^ mice; however, it remains possible that adaptive changes occurred as a result of the loss of CD73 in CD73^−/−^ tissues and these may underlie the different effects of TNAP-I between the two genotypes.

The results reported here are in contrast to our previous findings with cultured rat cortical neurons, astrocytes and co-cultures of neurons and astrocytes [18], [25] in which we found that astrocytes and co-cultures, but not neuron cultures, produced extracellular adenosine via a mechanism that was inhibited by AOPCP. Similarly, several studies report that CD73 is important for regulating adenosine levels in peripheral tissues during hypoxia and inflammation [11], [13], [26]. These studies led us to propose that ATP, released as a gliotransmitter, was an important source of adenosine during basal conditions, hypoxia/ischemia and treatment with the ionotropic glutamate receptor agonist N-methyl-D-aspartate. It has previously been reported that the gliotransmitter ATP is important for adenosine production during basal conditions and theta burst stimulation used to produce long term potentiation, but not during hypoxic conditions [27], [28]. It has also been reported that adenosine is released directly and not produced from extracellular ATP [29]–[32]. Thus, while several lines of investigation indicate that cellular release of ATP and CD73 activity are important for producing extracellular adenosine, other mechanisms for adenosine regulation are also evident. From the studies reported here, we conclude that CD73 is not a key enzyme for regulating extracellular adenosine levels, or adenosine A_1_ receptor activity, in basal or hypoxic/ischemic conditions in hippocampal slices during low frequency stimulation.

Previously, we reported that neuronal ENTs are more important for adenosine uptake than adenosine release during low frequency stimulation of normoxic, hypoxic or ischemic hippocampal slices [9]. Interestingly, a recent report [32] concluded that ENTs were responsible for adenosine release from hippocampal CA1 neurons during high frequency stimulation. These experiments used intraneuronal delivery of inosine to competitively inhibit ENT-mediated adenosine release and attenuate the inhibitory effect of adenosine at its receptors. Further research is required to reconcile these contrasting findings.

In conclusion, this study shows that CD73 is active in brain of Wt mice but the absence of CD73 does not reduce the activation of adenosine receptors that occurs in response to exogenous application of ATP or in response to hypoxia or OGD. TNAP activity was only evident when CD73 was absent and does not appear to provide a significant contribution to adenosine production in Wt mice. Whether another enzyme, perhaps restricted to the membrane microdomains containing adenosine A_1_ receptors, contributes to adenosine production in physiological and pathological conditions remains to be determined.
